# The effects of prebiotics on gastrointestinal side effects of metformin in youth: A pilot randomized control trial in youth-onset type 2 diabetes

**DOI:** 10.3389/fendo.2023.1125187

**Published:** 2023-02-23

**Authors:** Sydney A. Dixon, Sidharth Mishra, Katrina B. Dietsche, Shalini Jain, Lilian Mabundo, Michael Stagliano, Andrea Krenek, Amber Courville, Shanna Yang, Sara A. Turner, Abby G. Meyers, Doris E. Estrada, Hariom Yadav, Stephanie T. Chung

**Affiliations:** ^1^ National Institute of Diabetes & Digestive & Kidney Diseases (NIDDK), National Institutes of Health, Bethesda, MD, United States; ^2^ USF Center for Microbiome Research, Microbiomes Institute, University of South Florida Morsani College of Medicine, Tampa, FL, United States; ^3^ Department of Neurosurgery and Brain Repair, University of South Florida Morsani College of Medicine, Tampa, FL, United States; ^4^ Nutrition Department, Clinical Center, National Institutes of Health, Bethesda, MD, United States; ^5^ Children’s National Hospital (CNH), Washington, DC, United States

**Keywords:** diabetes, side effects (SE), gastrointestinal, microbiome, metformin, prebiotics, fiber, youth

## Abstract

**Disclosure summary:**

Dr. Yadav is Chief Scientific Officer and Co-Founder of Postbiotics Inc and has no conflict of interest with this work. All other authors have no conflicts of interest to disclose.

**Background:**

Metformin is the only approved first-line oral glucose lowering agent for youth with type 2 diabetes mellitus (Y-T2DM) but often causes gastrointestinal (GI) side effects, which may contribute to reduced treatment adherence and efficacy. Prebiotic intake may reduce metformin’s side effects by shifting microbiota composition and activity.

**Objective:**

The aims of this study were to determine the feasibility and tolerability of a prebiotic supplement to improve metformin-induced GI symptoms and explore the changes in glycemia and shifts in the microbiota diversity.

**Methods:**

In a two-phase pilot clinical trial, we compared, stool frequency and stool form every 1-2 days, and composite lower GI symptoms (weekly) at initiation of daily metformin combined with either a daily prebiotic or a placebo shake in a 1-week randomized double-blind crossover design (Phase 1), followed by a 1-month open-labeled extension (Phase 2). Plasma glycemic markers and stool samples were collected before and after each phase.

**Results:**

Six Y-T2DM (17.2 ± 1.7y (mean ± SD), 67% male, BMI (42 ± 9 kg/m^2^), HbA1c (6.4 ± 0.6%)) completed the intervention. Stool frequency, stool composition, and GI symptom scores did not differ by group or study phase. There were no serious or severe adverse events reported, and no differences in metabolic or glycemic markers. After one week Phase 1metformin/placebo *Proteobacteria*, *Enterobacteriaceae*, and *Enterobacteriales* were identified as candidate biomarkers of metformin effects. Principle coordinate analyses of beta diversity suggested that the metformin/prebiotic intervention was associated with distinct shifts in the microbiome signatures at one week and one month.

**Conclusion:**

Administration of a prebiotic fiber supplement during short-term metformin therapy was well tolerated in Y-T2DM and associated with modest shifts in microbial composition. This study provides a proof-of-concept for feasibility exploring prebiotic-metformin-microbiome interactions as a basis for adjunctive metformin therapy.

**Clinical trial registration:**

https://clinicaltrials.gov/, identifier NCT04209075.

## Background

Metformin is the most widely prescribed anti-diabetic agent in the world and a first line treatment for type 2 diabetes (T2DM) in both adults and children (1). However, metformin non-responsiveness is an important clinical challenge, occurring in 20-50% of youth and adults. Reduced treatment adherence may be multifactorial and is a well-recognized and potentially modifiable risk factor of non-responsiveness (2, 3). We and others have shown that medication-related gastrointestinal (GI) side effects (bloating, diarrhea, cramping, nausea, and vomiting) are a common barrier to metformin adherence and maximal dose escalation (4, 5). Side effects are observed in >80% of individuals newly initiated on metformin and ~10-30% of patients on long-term therapy with estimates of 1 in 4 youth taking metformin experiencing at least one GI side effect (5–7). Challenges are magnified in youth-onset T2DM (Y-T2DM), as metformin is the only oral medication that is approved for use in the 10-17 year age group and age-related factors, including pubertal-related differences in medication responsiveness and microbial signatures, may play a role in the elevated risk (8). Metformin-induced shifts in gut microbiota have been implicated in the occurrence of side effects (9), yet, there is a paucity of studies examining the mechanisms of metformin inducing GI side effects and ways to mitigate this burden in youth.

While the precise mechanism of metformin-induced GI side effects remains elusive, emerging data strongly suggest that certain dietary fibers or fiber supplements, including prebiotics which affect the microbiome, may benefit patients with diabetes (10, 11). Prebiotics are specific types of non-digestible carbohydrates that selectively stimulate the growth and activity of healthy host colonic microbiota, yielding potential benefits (12). These types of fiber may improve gut inflammation (13, 14) and metabolic profiles in patients with and without diabetes (15–17). However, prebiotic supplements—when used in isolation and at high doses—have variable effects and may worsen GI symptoms and increase flatulence, due to an increase in methane and hydrogen sulfate producing bacteria (18, 19). Prebiotic supplements combined with polyphenols —naturally occurring compounds metabolized by the short chain fatty acid (SCFA)-producing bacteria (e.g. acetogens)—decrease flatulence and side effects by promoting growth of acetogens and moderating overgrowth of methanogens and sulfate reducers (20). In a small study in adults with T2DM, a prebiotic agent (a complex of inulin, beta-glucan, and polyphenols from blueberry pomace) improved metformin tolerability and fasting glycemia (21). The prebiotic cocktail also improved glucose profiles in healthy adults with overweight and obesity but the study did not explore changes in gut microbiome composition (22). Further, the gut-based mechanisms by which prebiotics influence metformin-induced GI side effects remain to be elucidated. Importantly, it remains to be established whether using this supplement is feasible in Y-T2DM. Age and socio-demographic differences in Y-T2DM, compared to adults with T2DM, include differences in dietary fiber intake, distinct microbiome signatures, and variations in taste and texture preferences (23–26). We propose that together the prebiotic-polyphenol would promote and support SCFA-producing bacteria and limit the overgrowth of metformin-induced *Escherichia spp* that have been associated with virulence factors and hydrogen sulfide gas production (9), which contributes in gut disturbances including bloating.

This pilot study examined the use of a prebiotic with polyphenols as an adjunct to improving metformin tolerability and facilitating short-term dose escalation and explored the underlying gut-based mechanisms of metformin and fiber in Y-T2DM. Our primary objective was to compare GI symptoms at initiation of daily metformin therapy when used with a daily metformin/prebiotic agent versus a metformin/placebo agent. We hypothesized that the metformin/prebiotic agent would be associated with higher tolerability scores (a composite score of lower GI-related side effects and stool consistency) compared to the placebo. Additional exploratory aims included evaluating changes in glucose and insulin concentrations and changes in gut microbiota diversity and bacterial phylogenetic abundances after the daily metformin/prebiotic agent use in contrast to the placebo.

## Methods and materials

The Metformin Influences Gut Hormones in Youth (MIGHTY) studies were designed to evaluate the pathophysiology of Y-T2DM and metformin mechanisms of action. Y-T2DM subjects were recruited from and evaluated consecutively at the Metabolic Clinical Research Unit at the National Institutes of Health Clinical Center (NIH CC), Bethesda, MD, USA (ClinicalTrials.gov registration no. NCT04209075). The protocol was approved by the Institutional Review Board of the National Institute of Diabetes and Digestive and Kidney Diseases (NIDDK). Parents provided written informed consent and youth gave assent prior to enrollment. Nine (9) participants were screened and 6 enrolled between February 2020 and May 2021 (**Supplemental Figure S1**). Enrollment was prematurely halted in July 2021 because of the COVID-19 pandemic interruptions in prebiotic supply.

### Study design and participants

This was a pilot randomized double-blind crossover trial in Y-T2DM (**Supplementary Figure S1**). Youth aged 10–25 years, diagnosed with Y-T2DM by the American Diabetes Association criteria (27), Tanner stage IV or V, with Hemoglobin A1c (HbA1c) ≤8% were recruited to participate in this MIGHTY-Fiber Study. Exclusion criteria included: positive diabetes related autoantibodies (GAD-65 and IA-2 autoantibodies); consumption of ≥ 2 or more servings of ≥6 oz of yogurt per day; chronic GI disease; gastric bypass surgery; cancer diagnosis or auto-immune disease; chronic insulin therapy within 3 months of the study; or use of antibiotics, immunosuppressants, hormonal contraceptives, lipid-lowering agents, proton-pump inhibitors, supraphysiologic systemic steroids, cholesterol medications, prebiotics, or probiotics in the previous month at time of screening.

### Study timeline and protocol

The study timeline and protocol are presented in **Supplementary Figure S2**. After screening, if the participants were on metformin, they discontinued their metformin therapy for a 7 day washout period., Participants wore the Dexcom G6^®^ continuous glucose monitor (CGM) for glycemic management and monitoring. This study was conducted in two phases: Phase 1 (a randomized double-blind crossover trial) and Phase 2 (an open-label extension). Weekly GI symptom questionnaires were completed to assess metformin tolerability.

Phase 1 consisted of two interventions including 1) metformin/prebiotic supplementation BiomeBliss^®^, and 2) metformin/placebo, with 5 study visits over 5 weeks. Participants were randomized to either intervention during Period 1 or Period 2. Following Period 1, participants underwent a two week washout based on literature documenting microbiome changes occurring within 24 hours (28) and to exceed >5 half-lives off metformin elimination (6-8 hours, and in the erythrocytes is 23 hours (29).

Phase 2 was an open label 4-week extension of metformin/prebiotic supplementation during which all participants were asked to continue taking metformin (850mg) with the prebiotic shake twice daily. After one month (visit 6), participants were evaluated with a protocol that was identical to visits 3 and 5.

During Periods 1 and 2, participants were provided with prepared pack-out meals with controlled macronutrient content and dietary fiber. The energy provided was based on estimated energy needs using the Mifflin St Jeor equation and a standard activity factor of 1.3 (30) with the goal of weight maintenance during the study. Menus were individualized to the participant’s food preferences and aimed to meet a macronutrient distribution of 15% protein, 35% fat, and 50% carbohydrate. Fiber content was not controlled across participants but was consistent within participants for Period 1 and Period 2 based on food record. Menu items avoided dietary sources of probiotics (yogurt) and non-nutritive sweeteners. Pack-out meals were not provided during Phase 2.

### Study medication, randomization, and blinding

Participants were randomized to the prebiotic or a placebo shake to be administered with metformin (850mg tablets) prior to visit 2 (**Supplemental Figure S2**). Three study agents were used: metformin standard release 850mg oral tablet, BiomeBliss^®^ powder, and placebo powder. Metformin 850 mg tablets were used within the approved dosing regimens as follows: at the start of each period (visit 2/4), participants took metformin 850mg once daily x 3 days, and the dose increased to 850mg twice daily for the remainder of the study period. The placebo or prebiotic supplement was dispensed as packets, for which participants received 1 packet once daily x 3 days and 1 packet twice daily for the remainder of the study period. All study medications were taken together. The macronutrient composition of the prebiotic supplement and placebo composition are illustrated in **Supplemental Table S1**. Study randomization was performed by an independent statistician with 1:1 allocation ratio. Blinding of the investigators and participants was maintained throughout the study. Medication adherence was determined by co-author (LM) who adjudicated pill and sachet counts at each visit.

### Study procedures

#### GI symptom questionnaires

We conducted ecological momentary assessments of GI symptoms, stool frequency, and King’s Stool Chart (21, 31) *via* mobile text messaging (**Supplemental Figure S2**). Stool consistency (not applicable, very hard, hard, formed, loose, watery), urgency to evacuate (no need to evacuate within 3 hours after dosing, need to evacuate within 3 hours, need to evacuate within 2 hours, need to evacuate within 1 hour), daily bowel movements (at least 1 movement every 3-4 days, at least 1 movement every 2 days, at least 1 movement per day, at least 2 movements per day), bloating sensation (not applicable, mild, moderate, severe), flatulence (less than normal, normal, moderately increased, greatly increased), and evacuation completeness (not applicable, incomplete, constipated) were assessed (**Supplemental Figure S3**) (21).

#### Stool collection and microbiome analysis

Stool was collected up to 24-hours prior to the visit or during visit 2, 3, 4, 5, or 6. Stool collected at home was stored in a sterile plastic vial at 4°C and processed immediately after receiving at NIH CC. Samples were flash frozen with liquid nitrogen and transferred to -80°C and stored until further analysis for microbiome sequencing and run in one batch. Fecal DNA was isolated for microbial compositional analysis using PowerSoil^®^ DNA Isolation Kit. Our well standardized 16S rRNA sequencing and bioinformatics pipelines were used to analyze gut microbiome signatures (32–34). In brief, universal primer pairs 515 F (barcoded) and 806 R, the bacterial V4 hypervariable region were used to amplify bacterial 16S rDNA (35). Amplified and uniquely barcoded amplicons were purified using AMPure^®^ magnetic purification beads (Agencourt, Beckman Coulter, CA, USA) and quantified in a Qubit-3 fluorimeter (InVitrogen, Carlsbad, CA, USA). The normalized amplicon library of concentration equal to 8pM was subjected for sequencing using Illumina MiSeq sequencer (using Miseq reagent kit v3) (35). Each sample bacterial sequences were de-multiplexed, quality filtered, clustered, and analysis were done by using base-space, R-based analytical tools, and quantitative insights into microbial ecology (QIIME) (32, 34).

#### Metabolites

Glucose and insulin were measured in plasma on the Cobas 6000 instrument (Roche Diagnostics, USA) using an enzymatic hexokinase assay or electrochemiluminescence, respectively. HbA1c was determined using the HPLC D10 instrument (Bio-Rad, USA). High sensitivity C-reactive protein (hsCRP) was measured in plasma with the immunoturbidometric method assay (Abbott Architect, USA). Fructosamine was measured in serum *via* colorimetric rate reaction (Roche Diagnostics, USA). Cholesterol, triglyceride and HDL cholesterol concentrations were measured by enzymatic assays (Abbott Architect, USA). LDL was calculated with the following equation:

LDL = (1.06*Chol) – (1.03*HDLC) – (0.117*Trig) – (0.00047*(TRIG*(Chol-HDLC))) + (0.000062*(Trig*Trig)) – 9.44

#### Continuous blood glucose monitoring

The Dexcom G6^®^ CGM was used for the duration of the study. Outcomes collected for each Period/Phase included percent time in range (70-180 mg/dL), percent wear time, mean glucose, glucose standard of deviation, and glucose coefficient of variation.

#### Activity and sleep monitoring

Average daily steps, waking and sleep time were quantified using a small, non-invasive, portable watch accelerometer (GT3X+ by Actigraph Inc., Pensacola FL) worn on the participant’s wrist.

#### Quality of life questionnaire

Participants completed PedsQL quality of life questionnaires at Visit 3, 5, and 6 (36).

#### Assessment of dietary intake

Three-day food records (2 weekdays and 1 weekend day) were completed prior to visit 2 and 6 and reviewed with the metabolic nutrition team (SY, ST) and coded into Nutrition Data Systems for Research (*NDSR*, Minneapolis, MN: University of Minnesota, version 2019) to estimate daily caloric and macronutrient intake. At visit 3 and 5, participants returned a daily checklist that was reviewed with the metabolic nutrition team to confirm what was consumed during the controlled *ad libitum* periods and that participants refrained from consuming other foods or beverages containing prebiotics or probiotics (e.g., yogurt, kefir, kombucha).

#### Metabolites and mixed meal test

Fasting plasma samples and a mixed meal tolerance test was conducted after a 10-12 hour fast. A liquid meal (50% carbohydrates, 33% fat, and 17% protein) was administered to provide ~30% of the estimated daily calorie requirements for weight maintenance determined by the Mifflin St. Jeor equation with an activity factor of 1.3 [30]. Blood samples were obtained at 0, 10, 20, 30, 40, 50, 60, 90, 120, 150, and 180 minutes to measure glucose and insulin concentrations.

#### Body composition

A dual X-ray absorptiometry (DXA) scan was performed once during visit 3 to measure fat mass and lean body mass.

### Statistical analysis

The primary outcome was the composite tolerability score based on four (4) GI symptom profile categories (stool consistency, urgency to evacuate, bloating sensation, and flatulence) over 1 week. The composite score of tolerability was constructed using the principal component analysis (PCA) based on the 4 GI side effect profile categories. PCA was used to account for the expected high inter-patient variability. Comparison of mean tolerability scores over 1 week was analyzed by linear mixed models, adjusting for baseline score. Pre-specified covariates were treatment period and sequence effects from the crossover design. Demographic and exploratory metabolic variables were analyzed with repeated measures analysis of variance accounting for sequence and period effects. Statistical analyses were performed using SAS (PCA analysis) and STATA (version 17.1; Stata Corp, College Station, TX).

For the microbial analysis, the Kruskal–Wallis and Wilcoxon signed-rank test, implemented among classes set to 0.01, were used to determine alpha-diversity indices. Differences in beta-diversity were determined using permutational multivariate analysis of variance (PERMANOVA), a permutation-based multivariate analysis of variance to a matrix of pairwise distance to partition the inter-group and intra-group distance. An unpaired two-tailed Student’s t-test was used to compare alpha-diversity indices and bacterial abundance between the two groups. The LEfSe (Linear discriminatory analysis [LDA] Effect Size) was used to identify unique bacterial taxa that drive differences between different study groups (37) and logarithmic LDA score cut-off was set to 3, and the strategy for multiclass analysis was set to “all-against-all”. All the statistically analyzed bar graphs are presented in the form of mean ± SEM. QIIME and R packages were used for statistical analyses.

## Results

### No change in side effects or glycemic markers with metformin/prebiotic

Youth participants (n=6) were 67% male and aged 17.2 ± 1.7 years with a mean baseline BMI of 42 ± 9 kg/m2, HbA1c 6.4 ± 0.6%, and were within 5 years of diagnosis of diabetes (**Table 1**). All participants were prescribed metformin therapy before trial initiation. Two of the six participants reported a history of non-adherence to metformin therapy because of diarrhea and bloating. Average total energy intake (placebo: 2353 ± 319 vs prebiotic: 2385 ± 775 kcal, *P*=0.936) and percent intake from carbohydrates and fiber (data not shown) did not differ in Phase 1. Dietary total intake and macronutrient composition were also similar between Phase 1 and 2 (Phase 2: 2262 ± 710 kcal). Metformin and prebiotic adherence throughout the study were 92 ± 16% and 90 ± 23%, respectively.

**Table 1:** Demographic and metabolic characteristics of participants.

Metabolic Characteristics	Baseline	Phase 1 Placebo	Phase 1 Prebiotic	Phase 2 Prebiotic	P-value
**Age, years**	17.2 ± 1.7				
**Male, n (%)**	4 (67)				
**Duration of diabetes (years)**	2.3 ± 1.6				
**Hemoglobin A1C (%)**	6.4 ± 0.6				
**Lean body mass, kg**	62.5 ± 8.7				
**Fat body mass, kg**	52.0 ± 12.2				
**Weight, kg**	125.0 ± 19.2	123.7 ± 19.8	124 ± 20.2	124.0 ± 21.2	0.18
**BMI, kg/m2**	41.3 ± 9.0	40.8 ± 8.6	41.2 ± 8.9	41.2 ± 9.4	0.24
**Systolic Blood Pressure, mmHg**	125 ± 10	126 ± 13	126 ± 12	124 ± 18	0.85
**Diastolic Blood Pressure, mmHg**	70 ± 8	70 ± 5	74 ± 7	70 ± 8	0.15
**Fructosamine mmol/L**		232 ± 32	235 ± 32	229 ± 45	0.45
**hsCRP, mg/L**		7.2 ± 9.1	7.6 ± 8.1	11.5 ± 16.2	0.37
**ESR, mm/hour**		18 ± 20	15 ± 14	15 ± 11	0.42
**Fasting LDL Cholesterol, mg/dL**		74 ± 28	69 ± 34	72 ± 30	0.16
**Fasting HDL Cholesterol, mg/dL**		38 ± 5	37 ± 5	34 ± 7	0.91
**Fasting Triglycerides, mg/dL**		48 (41, 137)	59 (51, 125)	54 (50, 88)	0.52
**Mixed Meal Tolerance Test**					
**Fasting glucose (mg/dL)**		110 ± 24	102 ± 15	109 ± 19	0.44
**Glucose AUC (mg/dL●min)**		27251 ± 6956	24274 ± 4229	27113 ± 6705	0.38
**Fasting insulin (uU/mL)**		36.2 (17.5, 64.5)	38.1 (14.8, 43.8)	31.5 (10.9, 66)	0.40
**Insulin AUC ( uU/mL●min)**		40658 ± 25385	33215± 14007	37789 ±16367	0.07
**Continuous Glucose monitor**					
**CGM Active (%)**	90 ± 12	90 ± 12	88 ± 16	85 ± 14	0.81
**Average Glucose (mg/dL)**	153 ± 25	135 ± 23	122 ± 26	136 ± 31	0.07
**Time in range (%)**	76 ± 20	92 ± 13	93 ± 11	85 ± 18	0.97
**Glucose SD (mg/dL)**	34 ± 14	24 ± 11	22 ± 13	28 ± 14	0.15
**Glucose CV (mg/dL)**	22 ± 7	17 ± 5	17 ±7	20 ± 6	0.87
**Daily Activity and Sleep (n=5)**					
**Waking (min/day)**		938 (915, 1025)	990 (917, 1009)	915 (910-945)	0.44
**Sleep minutes (min/day)**		354 (322, 372)	394 (375, 396)	332 (290-346)	0.15
**Steps (daily)**		8158 (6667, 8794)	8878 (8621, 9581)	9082 (7779, 10270)	0.34

**Figure 1:**
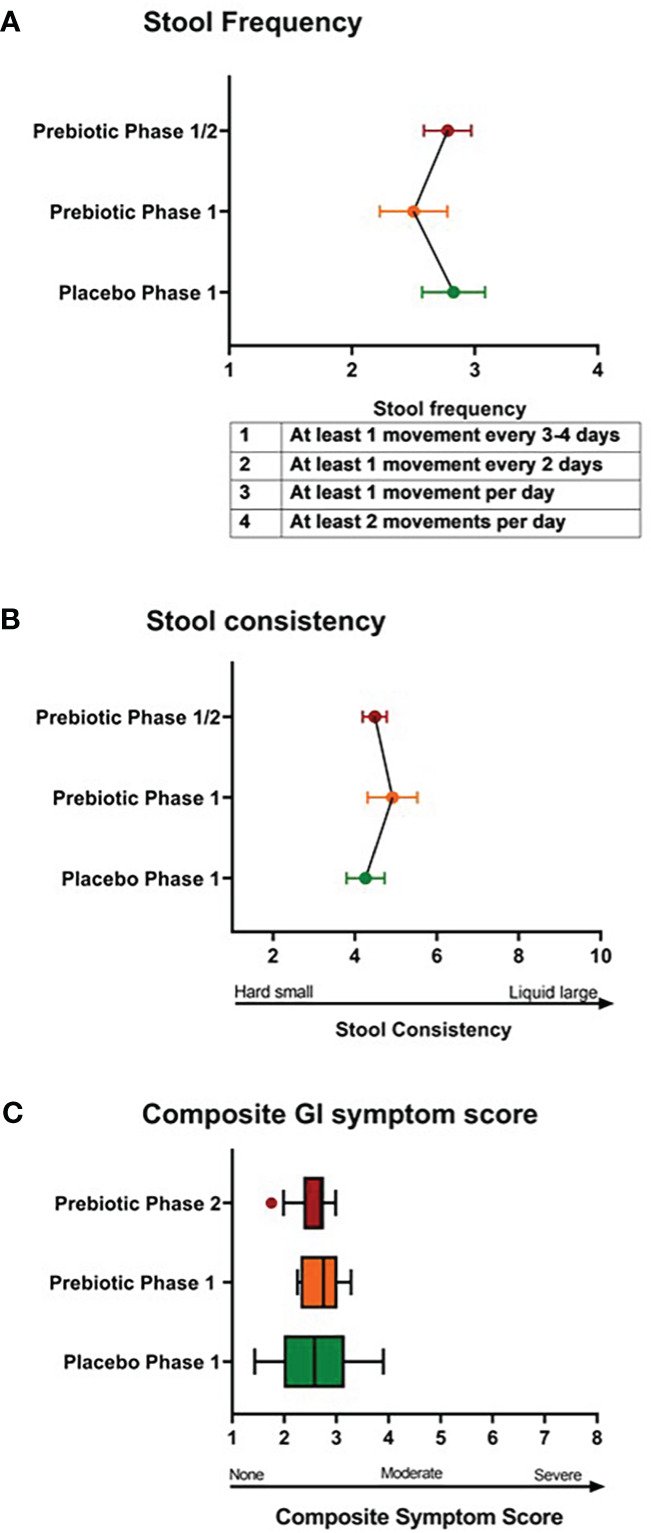
Comparison of stool frequency, consistency, and composite gastrointestinal symptoms. There were no differences in **(A)** stool frequency, **(B)** stool consistency, **(C)** composite gastrointestinal (GI) symptoms score between Phase 1 metformin/placebo green) vs. Phase 1 metformin/prebiotic (orange) vs. Phase 2 metformin/prebiotic (red). Comparisons between groups were made by linear mixed models, adjusting for baseline.

At baseline, youth had bowel movements every 1-2 days and soft-form stool. There were no differences in stool frequency, consistency, or composite GI symptom scores between one week Phase 1 metformin/prebiotic, Phase 1 metformin/placebo, or one month Phase 2 metformin/prebiotic (**Figure 1**). For the primary outcome of the composite score, there was no carryover effect, no difference between Phase 1 metformin/placebo and Phase 1 metformin/prebiotic (*P*=0.3243), and no difference between Phase 1 metformin/placebo and Phase 2 metformin/prebiotic (*P*=0.8257). Using the common terminology criteria for adverse events, CTCAE version 4 (38), grade 1 nausea was reported by one participant and grade 1 diarrhea by another participant (**Supplemental Table S2**). No adverse events were observed in Phase 2 and there were no moderate or severe adverse events (Grade 2 or higher) during the entire study (**Supplemental Table S2**). Quality of life did not differ by group or phase (data not shown).

Glycemic and metabolic variables during the mixed meal tolerance test and continuous glucose monitoring are shown in **Table 1**. There were no changes in overall glycemia (fructosamine or glucose AUC), markers of inflammation (hsCRP/ESR), or lipid panel markers in either the placebo/metformin or Phase 1 or Phase 2 prebiotic groups. There were no significant differences in CGM glycemic measures across period or phase, however there was a trend for lower mean average glucose with Phase 1 metformin/prebiotic supplementation (**Table 1**).

### Global changes in gut microbiome during phase 1 and 2

**Figure 2:**
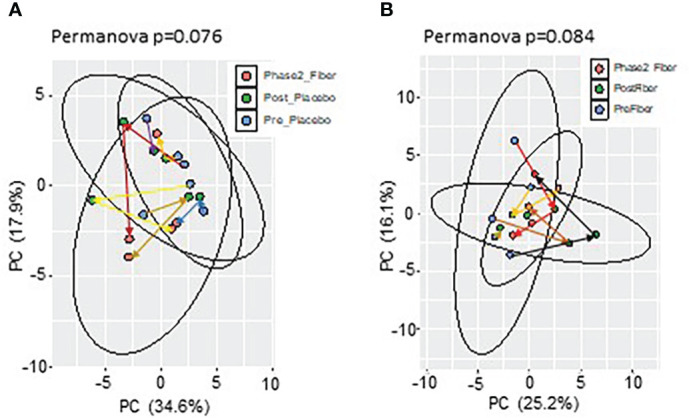
Principal component analysis of microbiome composition in Phase 1 and Phase 2. Trend for shifts in microbiome beta-diversity **(A)** between Pre_Placebo (pre metformin/placebo), Post_Placebo (post metformin/placebo) vs Phase2_Fiber (Phase 2 metformin/prebiotic) and **(B)** between Pre_Fiber (pre Phase 1 metformin/prebiotic), Post_Fiber (post Phase 1 metformin/prebiotic) vs Phase2_Fiber (post Phase 2 metformin/prebiotic).

To evaluate metformin and prebiotic mediated effects on the gut microbiome, we explored changes in beta diversity throughout the course of the study. Principal coordinate analyses revealed that during Phase 1 and Phase 2 there were overall trends for shifts in beta diversity (**Figures 2A, B**). These results suggest that there are distinct shifts in the microbiome signature when metformin is administered in combination with placebo or in combination with prebiotic supplementation in Y-T2DM participants.

### One week phase 1 metformin/placebo associated with shifts in gut microbiome

**Figure 3:**
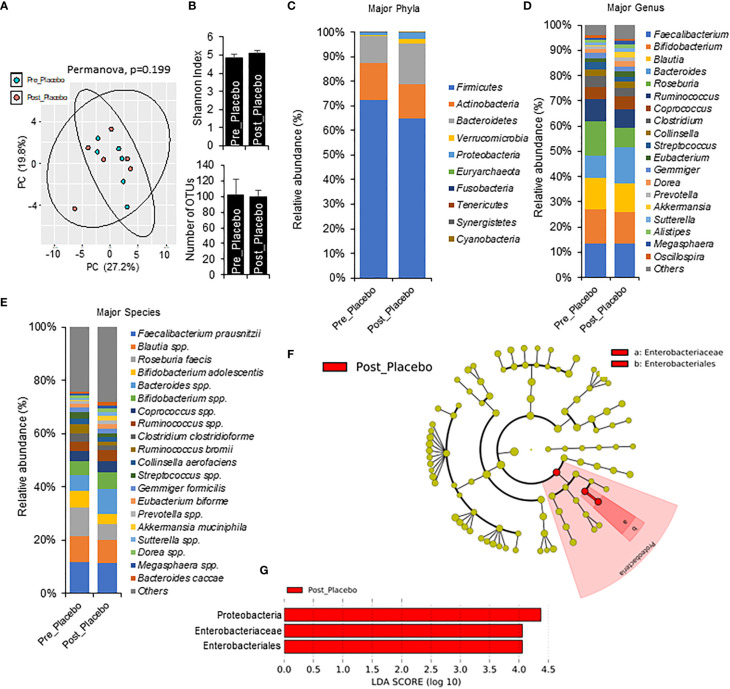
Microbiome signatures in pre metformin/place compared to Phase 1 Metformin/Placebo. **(A, B)** Principal component analysis of beta-diversity and alpha-diversity measure, Shannon Index, and number of OTUs did not differ between Pre_Placebo (pre metformin/placebo) to Post_Placebo (Phase 1 metformin/placebo). **(C–E)**There were modest changes in abundance of major phyla, genus, and species differs between Pre_Placebo and Post_Placebo. **(F, G)** LEfSe (Linear discriminant analysis Effect Size) analysis showed unique biomarkers in Pre_Placebo vs Post_Placebo. Data are mean and standard error of mean. Microbiome beta-diversity was assessed using the Bray-Curtis dissimilarity index and visualized with principal component analysis. The alpha-diversity indices and bacterial proportions were compared using the Kruskal-Wallis test followed by the Mann-Whitney multiple pairwise comparison test.

To understand more specific metformin-induced changes in the microbiome, we compared pre-metformin/placebo to one week of Phase 1 metformin/placebo (**Figure 3**). One week of metformin/placebo induced marginal shifts in the principal coordinate analysis of beta-diversity (**Figure 3A**) and no significant change in alpha-diversity indices (Shannon index) or the number of operational taxonomic units (OTUs) (**Figure 3B**). Compared to pre-metformin/placebo, Phase 1 metformin/placebo was associated with trends for changes in phyla, genus, and species (**Figures 3C–E**). The abundance of *Firmicutes* decreased and *Bacteroidetes* and *Verrucomicrobia* increased (**Figure 3C**), *Bacteroides* increased and *Roseburia* decreased (**Figure 3D**), and *Akkermansia muciniphila* increased while *Roseburia faecis* and *Bifidobacterium adolescentis* decreased (**Figure 3E**) after Phase 1 metformin/placebo. LefSe analysis revealed unique changes in abundance of *Proteobacteria*, *Enterobacteriaceae*, and *Enterobacteriales*, as biomarkers of metformin/placebo effects (**Figures 3F, G**).

### One week phase 1 metformin/prebiotic associated with shifts in gut microbiome

**Figure 4:**
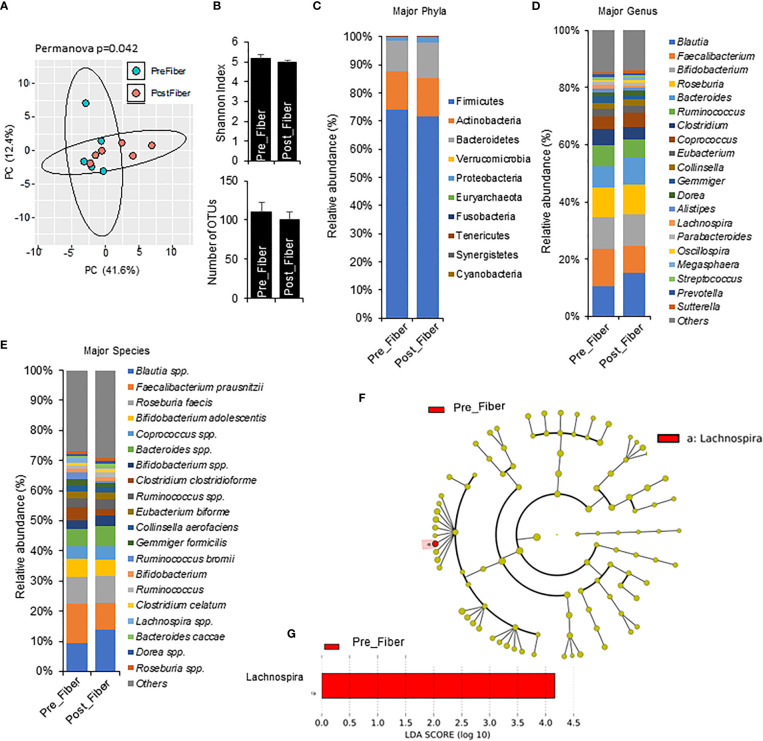
Microbiome signatures in pre metformin/prebiotic and Phase 1 metformin/prebiotic. **(A)** Principal component analysis of beta-diversity shows that microbiota composition differed between Pre_Fiber (pre Phase 1 metformin/prebiotic) and Post_Fiber (phase 1 metformin/prebiotic). **(B)** The alpha-diversity measure, Shannon Index and number of OTUs did not differ between groups. **(C–E)** The abundance of major phyla, genus, and species show marginal differences between Pre_Fiber and Post_Fiber. **(F, G)** LEfSe (Linear discriminant analysis Effect Size) analysis showed unique biomarkers in Pre_Fiber vs Post_Fiber. Data are mean and standard error of mean. Microbiome beta-diversity was assessed using the Bray-Curtis dissimilarity index and visualized with principal component analysis. The alpha-diversity indices and bacterial proportions were compared using the Kruskal-Wallis test followed by the Mann-Whitney multiple pairwise comparison test.

To explore short term effects of prebiotic and metformin on the gut microbiota, we compared pre-metformin/prebiotic to one week of Phase 1 metformin/prebiotic. Phase 1 metformin/prebiotic altered microbiome beta-diversity; however, alpha-diversity and number of OTUs remained unchanged (**Figures 4A, B**). There were also trends for changes in phyla, genus, and species (**Figures 4C–E**). Marginal changes were detected in the relative abundance with an an increase in *Bacteroidetes* and a decrease in *Firmicutes* (**Figure 4C**), increased *Blautia* and decreased *Faecalibacterium* and *Lachnospira*, (**Figure 4D**), increased *Blautia spp* and *Bifidobacterium spp*, and decreased *Faecalibacterium prausnitzi and Clostridium clostridioforme* (**Figure 4E**) after Phase 1 metformin/prebiotic. LefSe analyses revealed that only *Lachnospira* abundance decreased specific for Phase 1 metformin/prebiotic (**Figures 4F, G**).

### One month phase 2 prebiotic/metformin showed distinct gut microbiota shifts

**Figure 5:**
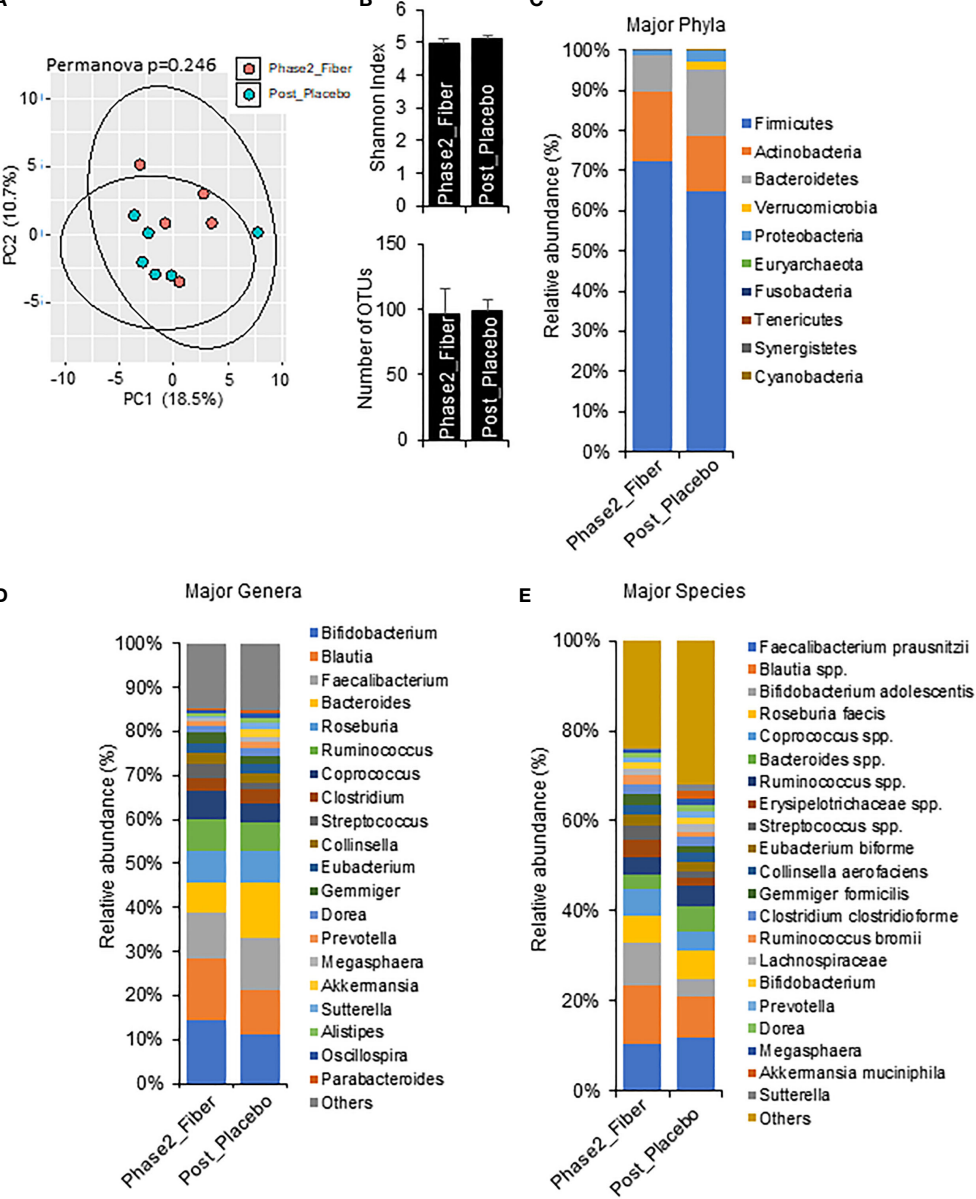
Microbiome signatures in Phase 2 prebiotic/metformin vs metformin/placebo. **(A, B)** Principal component analysis of beta-diversity and the alpha-diversity measure, Shannon Index and number of OTUs showed that microbiota composition was not different between Phase2_Fiber (Phase 2 prebiotic/metformin) and Post_Placebo (metformin/placebo). **(C–E)** The abundance of major phyla, genera, and species were similar in both groups. Data are mean and standard error of mean. Microbiome beta-diversity was assessed using the Bray-Curtis dissimilarity index and visualized with principal component analysis. The alpha-diversity indices and bacterial proportions were compared using the Kruskal-Wallis test followed by the Mann-Whitney multiple pairwise comparison test.

**Figure 6:**
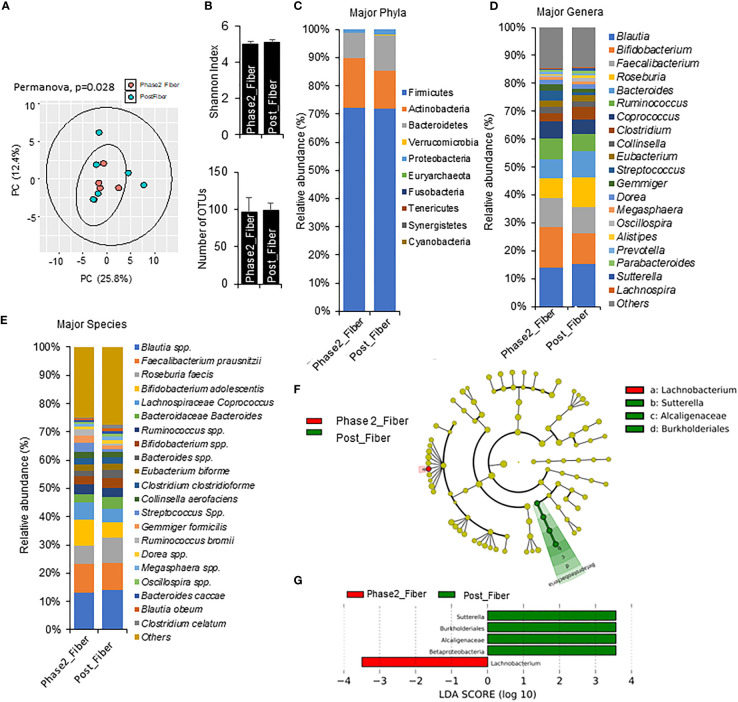
Microbiome signatures in Phase 2 prebiotic/metformin compared to Phase 1 prebiotic/metformin. **(A)** Principal component analysis of beta-diversity showed that microbiota composition differed between Phase2_Fibre (Phase 2 prebiotic/metformin) and Post_Fiber (Phase 1 prebiotic/metformin). **(B)** The α-diversity measure, Shannon Index and number of OTUs showed no differences between groups. **(C–E)** There were modest changes in abundance of major phyla, genus, and species. **(F, G)** LEfSe (Linear discriminant analysis Effect Size) analysis in Phase2_Fiber vs Post_Fiber showed unique biomarkers. Data are mean and standard error of mean. Microbiome beta-diversity was assessed using the Bray-Curtis dissimilarity index and visualized with principal component analysis. The α-diversity indices and bacterial proportions were compared using the Kruskal-Wallis test followed by the Mann-Whitney multiple pairwise comparison test.

To explore the changes with longer interventions of prebiotic and metformin use in a real-world setting, we compared one month Phase 2 metformin/prebiotic to one week Phase 1 metformin/placebo (**Figure 5**) and one week Phase 1 metformin/prebiotic (**Figure 6**). Compared to metformin/placebo, the Phase 2 metformin/prebiotic intervention was not associated with significant differences in beta or alpha-diversity (**Figures 5A, B**). There were trends of a modest increase in Firmicutes, a decreased Bacteroides (**Figure 5C**), no change in genus (**Figure 5D**), and an increased abundance of *Bifidobacterium* spp and *Blautia* spp (**Figure 5E**).

Compared to Phase 1 metformin/prebiotic, the Phase 2 metformin/prebiotic was associated with changes in microbiome beta-diversity but not alpha-diversity (**Figures 6A, B**). There were incremental increases in Actinobacteria (**Figure 6C**), with trends for increased abundance of *Bifidobacterium*, decreased abundance of *Roseburia, Bacteroides*, (**Figure 6D**), increased *Bifidobacterium adolescentis*, and decreased *Roseburia faecis spp* after Phase 2 metformin/prebiotic (**Figure 6E**). Further, LefSe analyses revealed *Sutterella*, *Burkholderiales* and *Alcaligenaceae* were uniquely enriched and *Lachnobacterium* were uniquely suppressed after Phase 2 metformin/prebiotic (**Figures 6F, G**).

## Discussion

Metformin intolerance is an important clinical barrier to care and a potentially modifiable target for adjunctive treatment in Y-T2DM. This novel pilot study evaluated the tolerability and feasibility of using a prebiotic supplement and microbiome modulator at the time of metformin treatment initiation and dose escalation. The prebiotic supplement was feasible and well tolerated in youth and was not associated with increased GI symptoms or adverse reactions. Participants tolerated the rapid metformin dose escalation without adverse events. Our findings are consistent with studies in adults supporting prebiotics as adjunctive therapy in adults on metformin pharmacotherapy (21) (22). This study provided the proof-of-concept needed to further explore prebiotic dietary supplements as adjunctive management with metformin in a vulnerable population of youth with T2DM who have high rates of metformin failure associated with GI symptoms (5).

Prebiotics and dietary adjuncts to traditional medicines in Y-T2DM are attractive candidates for addressing the complex multi-faceted care considerations in youth, with studies already suggesting beneficial glycemic effects when combining prebiotics and polyphenols with metformin in adults with T2DM (39). Dietary prebiotic fiber is safe, relatively inexpensive, and may promote optimal cardiometabolic health, but is often under-consumed by adolescents in the United States (40). Prebiotic supplementation in this trial was designed to provide ~40% of recommended daily fiber intake value. Prebiotics exert their beneficial effects as they promote the growth and/or activity of SCFA-producing bacteria that may improve gut health (12, 19). In addition to the direct fiber-related metabolic effects to reduce cholesterol absorption and increase colonic transit time, prebiotics may also improve intestinal permeability and gut inflammation – two factors closely linked to obesity and T2DM (13, 14). However, excessive prebiotic use may increase carbon dioxide and hydrogen sulfide gas production (13). To balance the prebiotic effect, we employed a supplement of prebiotics and polyphenols –to preferentially promote acetate-producing bacteria growth and SCFA production and minimize methane- and hydrogen sulfate-producing bacteria (16, 17).

Notably, this study filled an important knowledge gap by demonstrating, for the first time in Y-T2DM, distinct metformin-induced shifts in gut microbiota signatures after one week of monotherapy or in combination with prebiotics. These findings extend previous studies in adults indicating metformin-induced shifts in microbiota occurred within 24 hours of drug initiation (21, 41). We demonstrated that metformin/placebo was associated with increases in SCFA-producing bacteria (*Akkermansia muciniphila*), findings that are consistent with the growing evidence supporting gut-based modulation as important mechanisms of metformin action (9, 41–44). We further identified that an increased abundance of *Proteobacteria*, *Enterobacteriaceae*, and *Enterobacteriales* were candidate biomarkers of metformin effects in Y-T2DM. With more research, these bacteria could be implicated as early biomarkers of metformin response.

The short term combination of metformin/prebiotic supplementation also resulted in potentially beneficial microbial shifts towards greater enrichment in some SCFA producing bacteria such as *Bifidobacterium adolescentis, Blautia*, and *Acintobacter*, but a decrease in others *(Firmucutes* and *Roseburia* spp). Notably, the addition of prebiotics to metformin therapy prevented increased abundance of *Enterobacteriaceae*, the family associated with *Escherichia spp* which are linked with metformin-associated GI side effects. These findings, while suggestive of a beneficial shift in microbiota with use of prebiotics and metformin, were limited and not directly associated with improvements in side effects or glycemia. Overall, the unique enrichments illustrated by the cladograms of metformin monotherapy and metformin/prebiotic supplement may be useful biomarkers of treatment response in future microbiome studies in youth on metformin therapy.

These foundational findings support the design of larger studies to evaluate whether the shifts in microbiome could be associated with metabolic improvement in Y-T2DM. Incremental trends for improved glucose and triglyceride homeostasis were observed during Phase 1 of our trial and abolished during the Phase 2 open-label period. A strength of this study, therefore, was to demonstrate that detailed metabolic phenotyping combining ecological momentary assessments of glycemia (CGM) and standardized mixed meal tests, were useful for identifying targets for metabolic phenotyping. The rigorous double-blind crossover design and controlled feeding periods during Phase 1 reduced the chances of carry-over effects in metformin, dietary intake, and prebiotics. The run-in and washout periods exceeded the five half-lives needed to eliminate metformin from the plasma and red blood cell compartments and minimized the carry-over effects of metformin and prebiotic changes in the microbiota. The cross-over study design also accounted for interindividual heterogeneity in microbiome signatures and host-environmental effects, increasing the ability to identify small microbiota shifts.

Generalizability of this pilot study was restricted by the small sample size with limited recruitment and product availability secondary to COVID-19 pandemic 2020-2022. Participants also had few GI symptoms at baseline and additional studies will be needed to determine the effectiveness in youth with increased frequency and/or severity of GI symptoms. Other limitations include multiple exploratory metabolic and microbiome analyses with a lack of correlation with stool metabolites such as short-chain fatty acids. Additionally, metformin treatment is associated with reduced lipopolysaccharide (LPS) and improvements in metabolic endotoxemia (45), but these were not measured in this study and could be important for assessing response in future analyses. Lastly, this pilot feasibility study was not designed to determine whether specific shifts in microbiome signatures would be associated with an improved metabolic profile. Rather, these data provide estimates of effect sizes for larger clinical trials using prebiotic-based supplements in youth.

## Conclusions

Metformin-induced side effects are an important clinical problem in Y-T2DM. This innovative study found that adjunctive prebiotic treatment was well tolerated and facilitated timely dose escalation without inducing GI side effects. Metformin alone and the prebiotic-metformin combination resulted in unique shifts in the beta-diversity of the microbiome that were detectable under controlled feeding conditions and in the free-living environment. Prebiotics should be considered in larger trials to evaluate their effectiveness in mitigating GI side effects and improving metabolisms and quality of life in a broader population of youth.

## Data availability statement

The data presented in the study are deposited in the BioProject repository, accession number PRJNA912677.

## Ethics statement

The studies involving human participants were reviewed and approved by Institutional Review Board of the National Institute of Diabetes & Digestive & Kidney Diseases. Written informed consent to participate in this study was provided by the participants’ legal guardian/next of kin.

## Author contributions

SC conceptualized and designed the study, recruited, and collected the data, conducted the analysis, and wrote the manuscript. AM, HY conceptualized and designed the study, revised, and edited the manuscript. SJ, AK, SM, AC, DE, LM, MS, SD, KD, SY, and ST made substantial contributions to data collection and analysis, revising, and editing the manuscript. SC is the guarantor of this work and, as such, had full access to all data in the study and takes responsibility for the integrity of the data and the accuracy of the data analysis. All authors contributed to the article and approved the submitted version.
