# Supplementary material for: The effects of prebiotics on gastrointestinal side effects of metformin in youth: A pilot randomized control trial in youth-onset type 2 diabetes
Source: Front Endocrinol (Lausanne). 2023 Feb 23;14:1125187. doi: 10.3389/fendo.2023.1125187 (PMC9996666; doi:10.3389/fendo.2023.1125187)
Supplement: Supplementary file 1 [file DataSheet_1.docx]

# Supplemental Figure S1. Consort Participant Flow Diagram


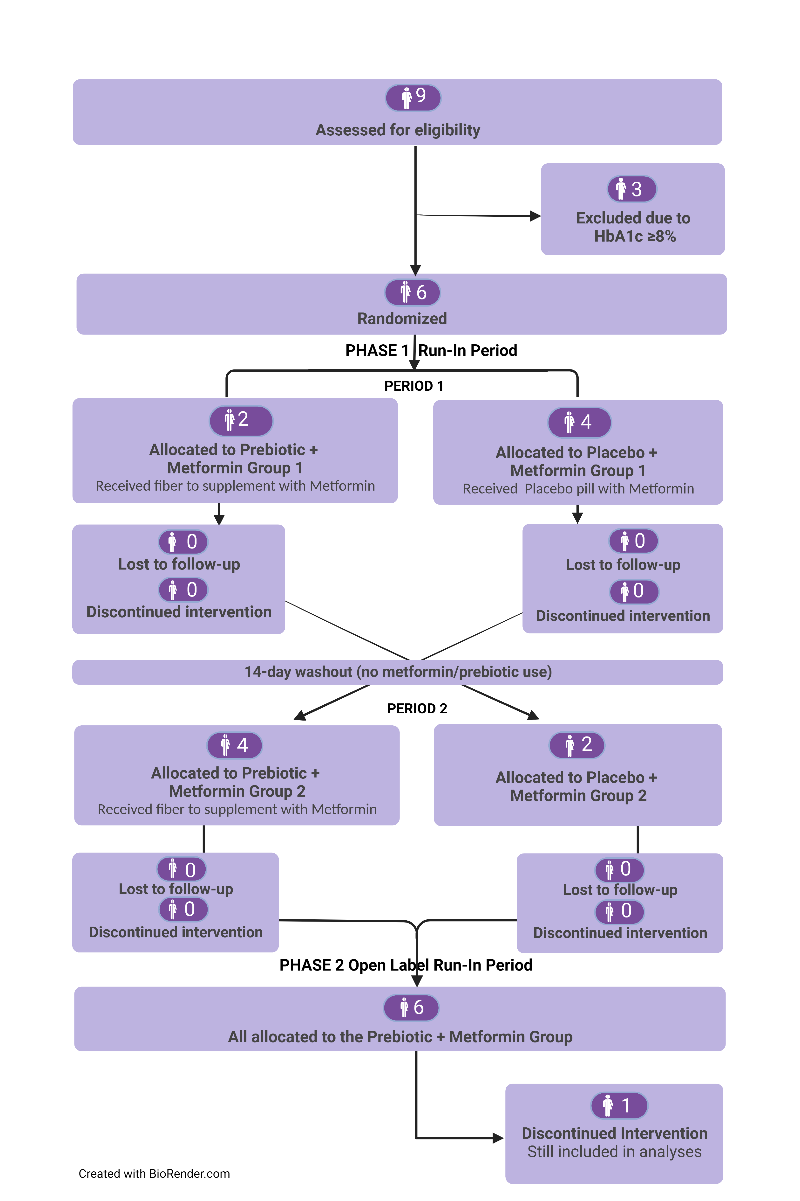


# Supplemental Figure S2. Study design and timeline

#
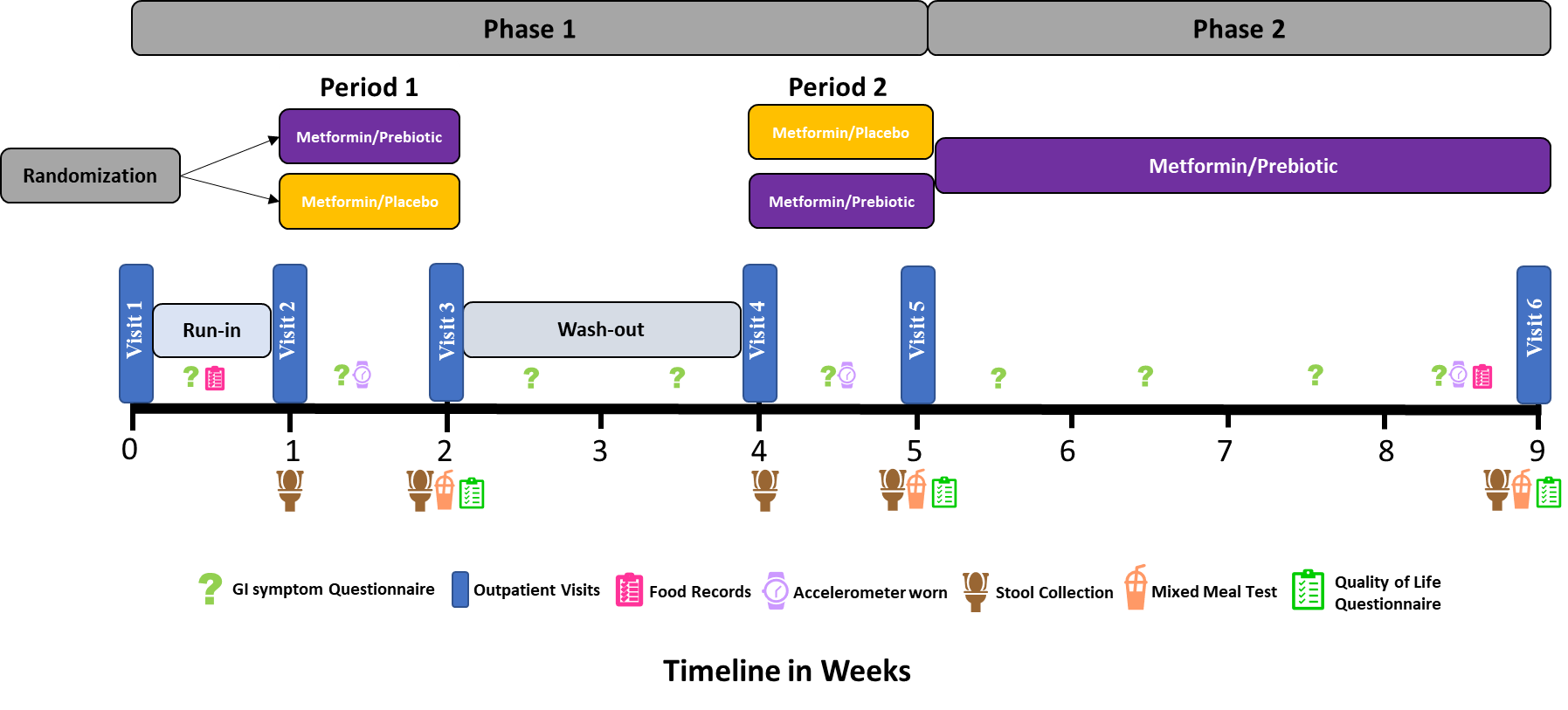


# Supplemental Figure S3. Sample GI Symptom Questionnaire in RedCap


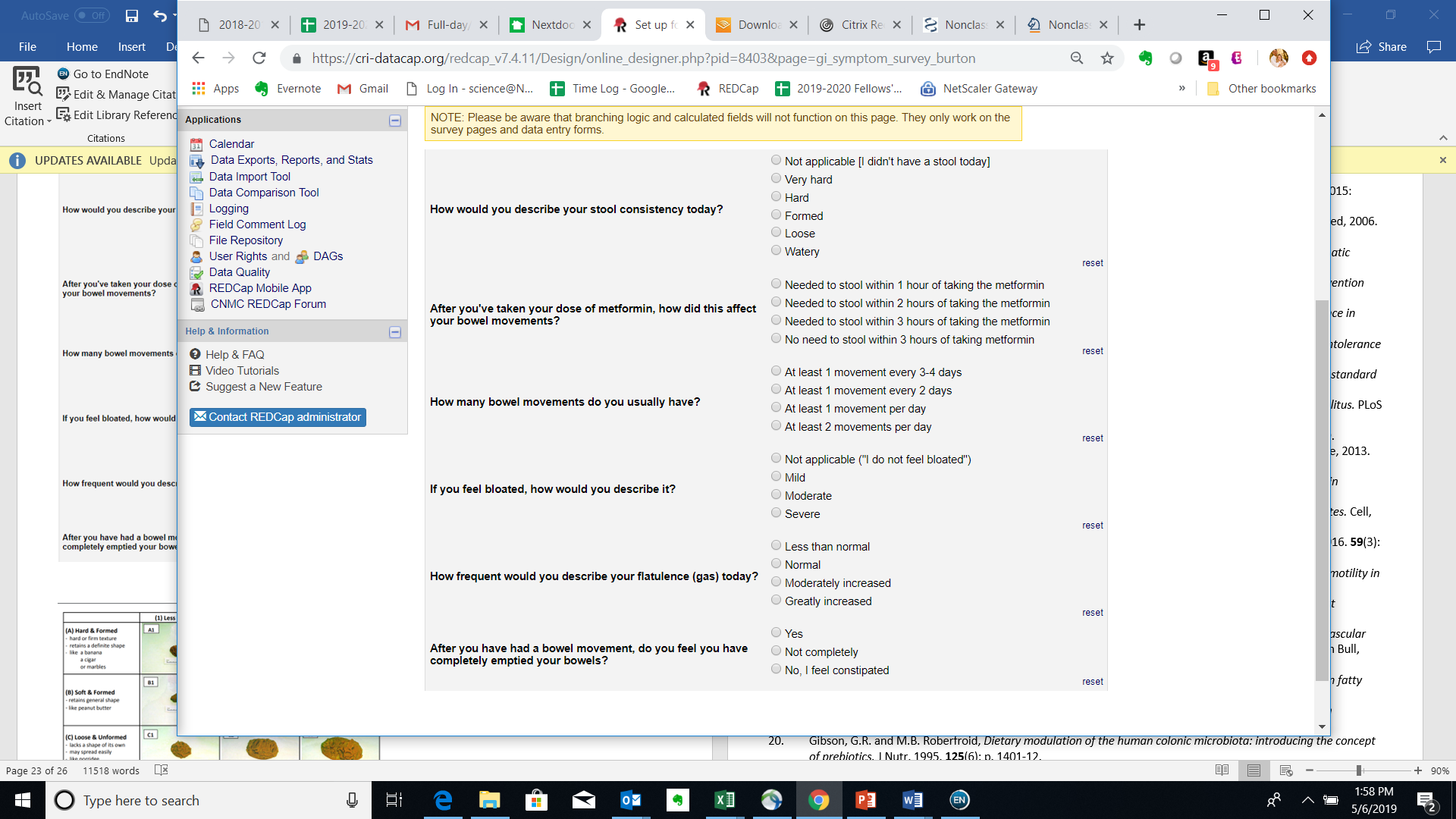


**Supplemental Table 1: Dietary nutrient composition of prebiotic supplement and placebo**

|  | **Biomebliss™** | **Gatorade G2®**  **(grape and arctic freeze)** |
| --- | --- | --- |
| **Calories per serving (kcal)** | 60 | 60 |
| **Grams per serving (g)** | 27 | 30 |
| **Ingredients** | inulin, blueberry extract, beta-glucan (oats), soy protein isolate, pomegranate flavor, xanthan gum, citric acid, stevia extract | sugar, citric acid, salt, sodium citrate, natural flavor, monopotassium phosphate, modified food starch, calcium silicate, sucralose, acesulfame potassium, blue 1 |
| **Fiber content (g)** | 9 | 0 |
| **Carbohydrate content (g)** | 22 | 16 |

**Supplemental Table 2. Adverse events categorized by CTCAE during the trial**

| Adverse Event | **Number of incidents in Phase 1 Placebo** | **Number of incidents in Phase 1 Metformin** | **Number of incidents in Open-label phase 2** | **Number of participants affected** |
| --- | --- | --- | --- | --- |
| Grade 1 Nausea | 1 | 1 | 0 | 1 |
| **Grade 1 Diarrhea** | 1 | 0 | 0 | 1 |
| **Grade 1 Hypoglycemia** | 0 | 1 | 0 | 1 |
| **Grade 1 Maculo-papular rash** | 0 | 1 | 0 | 1 |
| **Grade 2 or higher event** | 0 | 0 | 0 | 0 |

CTCAE: common terminology criteria for adverse events version 4, maintained by the National Cancer Institute. [Common Terminology Criteria for Adverse Events (CTCAE) | Protocol Development | CTEP (cancer.gov)](https://ctep.cancer.gov/protocolDevelopment/electronic_applications/ctc.htm) – last accessed 11.13.2022.

**Data Sharing and Availability**

Microbiome genomic data analyzed in this study was registered with the BioProject database. The BioProject ID PRJNA912677
